# Preliminary clinical assessment of dynamic carbon-11 methionine positron-emission tomography/computed tomography for the diagnosis of the pathologies in patients with musculoskeletal lesions: a prospective study

**DOI:** 10.1186/s41824-020-00083-x

**Published:** 2020-08-26

**Authors:** Takayoshi Shinya, Yoichi Otomi, Toshihiko Nishisho, Bettina Beuthien-Baumann, Michiko Kubo, Hideki Otsuka, Yoshimi Bando, Hiroaki Yanagawa, Koichi Sairyo, Masafumi Harada

**Affiliations:** 1 Department of Radiology, Tokushima University Hospital, 2-50-1, Kuramoto-cho, Tokushima City, Tokushima, 770-8503 Japan; 2 Division of Radiology, German Cancer Research Centre (DKFZ), Im Neuenheimer Feld 280, 69120 Heidelberg, Germany; 3 Department of Diagnostic and Therapeutic Radiology, Kawasaki Medical School General Medical Centre, 2-6-2 Nakasange, Kita-ku, Okayama City, Okayama, 700-8505 Japan; 4 Department of Orthopedics, Institute of Biomedical Sciences, Tokushima University Graduate School, 3-18-15 Kuramoto-cho, Tokushima City, Tokushima, 770-8503 Japan; 5 Department of Medical Imaging/Nuclear Medicine, Institute of Biomedical Sciences, Tokushima University Graduate School, 2-50-1, Kuramoto-cho, Tokushima City, Tokushima, 770-8503 Japan; 6 Division of Pathology, Tokushima University Hospital, 2-50-1, Kuramoto-cho, Tokushima City, Tokushima, 770-8503 Japan; 7 Clinical Trial Center for Developmental Therapeutics, Tokushima University Hospital, 2 Kuramoto-cho, Tokushima City, Tokushima 770-8503 Japan

**Keywords:** Musculoskeletal lesion, Sarcoma, Malignant lymphoma, Diagnostic validity, C-11 MET, Dynamic PET/CT, SUVmax

## Abstract

**Background:**

This study prospectively assessed the diagnostic capacity of dynamic carbon-11 methionine (C-11 MET) positron-emission tomography (PET)/computed tomography for the diagnosis of pathologies in patients with primary unknown musculoskeletal lesions (MSLs). In total, 13 patients with MSLs underwent dynamic scans (5–10 [phase 1], 10–15 [phase 2], 15–20 [phase 3], 20–25 [phase 4], 25–30 [phase 5], and 30–35 [phase 6] min post-injection of C-11 MET). We statistically compared the maximum standardised uptake values (SUVmax) and corresponding retention index for dynamic scans (RI-SUV) for five benign MSLs (BMSLs), five primary malignant musculoskeletal tumours (PMMSTs), four metastatic musculoskeletal tumours (MMSTs), and three malignant lymphoma (ML) cases and explored their diagnostic capacities using receiver operating characteristic (ROC) curve analyses.

**Results:**

SUVmax gradually decreased or remained similar with minimal fluctuations in all BMSL cases and four of five PMMST cases. In contrast, SUVmax increased over time in one case of PMMST and in all cases of MMST and ML. Significant differences were observed in SUVmax for all time phases and RI-SUV between BMSLs and MMSLs, in SUVmax for all time phases between PMMSTs and BMSLs, in SUVmax for all time phases and RI-SUV between non-PMMST-malignant tumours and BMSL, and in RI-SUV between non-PMMST-malignant tumours and PMMST. In ROC analyses, the areas under the curve yielded the highest values at 1.00 for differentiating most intergroup comparisons.

**Conclusions:**

Dynamic C-11 MET PET scans have the potential to be good predictors of discriminating MSLs in patients with primary unknown MSLs in clinical practice.

## Background

Musculoskeletal lesions (MSLs) constitute a wide range of physiological, biochemical, and genetic characteristics. In clinical practice, orthopaedic surgeons and radiologists encounter pathologically unknown MSLs. It is of major diagnostic importance to differentiate between a benign musculoskeletal lesion (BMSL) and malignancy from the aspects of indication for biopsy and determining optimal treatment. Moreover, differentiating MSLs with morphologic imaging such as conventional radiography, computed tomography (CT), and magnetic resonance imaging may result in the diagnostic dilemma of differential diagnosis due to non-specific features and considerable overlap with other pathologies in cases with atypical image findings (Fletcher et al., 2002; Raphael et al., 2013; Lim et al., 2013).

Functional imaging techniques, including thallium-201 (^201^Tl) scintigraphy, ^99m^Tc (V) dimercaptosuccinic acid scintigraphy, and fluorine-18-fluorodeoxyglucose (^18^F-FDG) positron emission tomography/computed tomography (PET/CT), are increasingly being used to obtain complementary information in specific clinical situations (Inai et al., 2015; Keller et al., 2017; Kobayashi et al., 1995; Choong et al., 2004; Shinya et al., 2015; Hicks, 2005). However, even with metabolic imaging including ^18^F-FDG PET/CT, there are overlapping features among MSLs. One limitation is the relatively high rate of false-positive results caused by inflammatory tissues. Therefore, the development of post-^18^F-FDG molecular probes such as carbon-11 methionine (^11^C-MET), which reflects increased amino acid transport and protein synthesis and is related to cellular proliferation activity, is expected to overcome these problems (van Waarde et al., 2006).

Absolute PET quantification of physiological parameters via tracer kinetic modelling and dynamic PET imaging is expected to facilitate elucidation of the pathophysiological mechanisms of histology and extraction of physiological or biochemical parameters via tracer kinetics (Dimitrakopoulou-Strauss et al., 2001; Dimitrakopoulou-Strauss et al., 2002; Rusten et al., 2013; Okazumi et al., 2009; Dimitrakopoulou-Strauss et al., 2010a; Dimitrakopoulou-Strauss et al., 2010b). Despite its availability, dynamic PET imaging with kinetic model analysis has been primarily confined to research centres and has not been used in busy clinical settings, mainly due to time-demanding imaging protocols and sophisticated evaluation tools for the evaluation of dynamic scans (Kwee et al., 2010). Nevertheless, technical developments in dynamic ^18^F-FDG PET/CT have enabled the investigation of perfusion-dependent FDG uptake and metabolic activity in various tumour entities (Schierz et al., 2013; Bernstine et al., 2011; Epelbaum et al., 2013; Belakhlef et al., 2012; Nakajima et al., 2016). Recent studies have performed dynamic PET/CT in list-mode without kinetic model analysis as a simple method to predict pathological and clinicopathological findings of tumours (Schierz et al., 2013; Nakajima et al., 2016; Shinya et al., 2019; Shinya et al., 2020). In a study using small animal PET analysis, Zhao et al. demonstrated that the dynamic pattern of ^11^C-MET uptake was significantly different between granulomas and tumours (Zhao et al., 2011). In another paper on brain tumours, dynamic changes in ^11^C-MET uptake varied between different tumour pathologies, and ^11^C-MET PET imaging revealed additional information to differentiate brain tumours (Aki et al., 2012). However, to our knowledge, no study to date has assessed the diagnostic capacity of dynamic ^11^C-MET PET/CT for differentiating between BMSL and malignant musculoskeletal lesions (MMSLs) and for differential diagnosis in MSL.

This prospective study assessed the transitional patterns of ^11^C-MET uptake in each type of MSL, which include BMSLs, primary malignant musculoskeletal tumours (PMMSTs), metastatic musculoskeletal tumours (MMSTs), and malignant lymphomas (MLs), on dynamic scans and investigated the diagnostic capacity of dynamic ^11^C-MET PET/CT with list-mode for the differentiation of patients with primary unknown MSLs.

## Methods

### Study population

This single-centre prospective study was approved by our institutional review board and ethics committee. A written informed consent form was signed by all patients. All procedures for studies involving human participants were conducted in accordance with the 1964 Helsinki Declaration and its later amendments or comparable ethical standards. We included patients older than 20 years with pathologically unknown MSL, in whom diagnosis was finally determined on pathologic examination; patients who had not undergone therapy for MSL before PET/CT; and patients without malignancy within the last 5 years.

### ^11^C-Methionine PET/CT procedures

The PET study was performed according to standardised procedures. A dose of 3.7 MBq/kg MET was injected intravenously. PET/CT imaging was conducted using a single PET/CT system (Discovery PET/CT 710; GE Healthcare, Chicago, IL, USA), which had list-mode data acquisition and enabled four-dimensional data acquisition (i.e. dynamic studies). The protocol included the following two PET/CT acquisitions: (1) a dynamic PET acquisition (limited to the single bed position) and (2) a low-dose CT scan centred on the largest MSL of interest and initiated 5 min after the intravenous injection of ^11^C-MET. All participants underwent dynamic PET/CT examinations.

In the first acquisition, low-dose CT was acquired for PET attenuation correction, anatomical information, and image fusion. In the second acquisition, dynamic ^11^C-MET PET/CT imaging was conducted as list-mode continuous scanning with every measured value stored as raw data with exact time stamps beginning at 5 min after the MET bolus and continuing for 30 min. The dynamic series was acquired with the field of view over the lesions of interest (limited to a single bed position, with the coverage in the patient’s longitudinal direction of 15.042 cm). Patients were instructed to lie motionless for the duration of the dynamic study. We subsequently reconstructed the data as six frames at 300-s intervals. The list-mode files were reconstructed on the PET/CT scanner. The frames were reconstructed using three-dimensional attenuation-weighted ordered-subset expectation maximisation with two iterations and 16 subsets (VUE Point FX; GE Healthcare) with 4-mm post reconstruction Gaussian filter, attenuation image segmentation, and a 192 × 192 pixel matrix.

### Analysis of PET/CT images

The dynamic PET/CT images were reviewed by two nuclear medicine physicians with 19 and 17 years of experience, respectively. Both readers were blinded to all clinical, pathological, and other imaging findings. If the results of the two physicians differed, they discussed the findings until a consensus was reached. Any obvious foci of MET uptake within MSLs that were increased relative to the surrounding tissue that was unrelated to physiologic sites of tracer uptake were considered positive findings.

By using the PET/CT images, we carefully placed the volume of interest (VOI) on the MSL to exclude MET accumulation in normal tissue and large vessels. Circular VOIs were drawn to encompass each lesion contour on transaxial images. VOIs were placed by consensus between the two nuclear medicine physicians.

For semiquantitative analysis of MET uptake, we adopted the standardised uptake value (SUV). The SUVs were calculated using lean body mass, as follows:

SUV = concentration of radioactivity (Bq/kg) × [body mass (kg)/injected radioactivity (Bq)]

To minimise partial-volume effects, the SUVmax within the VOIs was used for the statistical analyses. The SUVmax was measured for each dynamic phase (i.e. SUV1, 5–10 min; SUV2, 10–15 min; SUV3, 15–20 min; SUV4, 20–25 min; SUV5, 25–30 min; and SUV6, 30–35 min). Furthermore, we calculated the RI-SUV from the SUVmax, based on the following formula:

Retention index of the phase SUVmax (RI ‐ SUV[%]) = (SUV6 – SUV1) × 100/SUV1

In addition, we calculated the mean SUV (SUVmean) for each subject.

The maximal diameter of each tumour lesion was measured on axial low-dose CT images.

### Statistical analysis

The Mann-Whitney *U* test or Kruskal-Wallis test followed by the Steel-Dwass test were employed to analyse the differences in SUVmax for each of the 6 dynamic phases and RI-SUV. If the statistical test revealed a significant difference in each index among the groups of MSLs, an additional receiver operating characteristic (ROC) analysis of the SUVmax and RI-SUV was conducted to evaluate the predictive performance of calculating each index for discriminating the groups. Discrimination was assessed by using the area under the ROC curve (AUC). We determined the optimal cut-off values that maximised the sensitivity and specificity for each index and the corresponding sensitivity, specificity, positive predictive value (PPV), negative predictive value (NPV), and accuracy. Significant differences between the two AUCs, as determined from the ROC curve, were assessed with Delong’s test. Statistical analyses were conducted using EZR version 1.37 (Saitama Medical Centre, Jichi Medical University, Saitama, Japan; available at www.jichi.ac.jp/saitama-sct/SaitamaHP.files/statmedEN.html) which is a graphical user interface for R (The R Foundation for Statistical Computing, Vienna, Austria) (Kanda, 2013). Statistical significance was set at *p* < 0.05.

## Results

### Patients and tumour characteristics

Patient and tumour characteristics are indicated in Table 1. Between February 2016 and November 2016, a total of 13 patients met the criteria for this prospective analysis and underwent dynamic ^11^C-MET PET/CT (nine males and four females; age [mean ± SD], 69.69 ± 10.23; range, 54–85 years). Seventeen lesions were interpreted as bone and soft tissue lesions on PET/CT evaluations (13 soft tissue, four bone). MSLs were classified according to the WHO Classification of Tumours of Soft Tissue and Bone, Fourth Edition, by pathologists blinded to PET results (Fletcher et al., 2013).

**Table 1:** Characteristics of patients and tumours

No./patient no./age/sex	Histology	Tumour site	Diameter (mm)
**A. Benign musculoskeletal lesion**			
1/1/80/male	Osteochondroma	Knee	20
2/2/68/male	Osteochondroma	Knee	40
3/3/67/male	Haematoma	Femur	245
4/4/77/male	Schwannoma	Axilla	56
5/5/61/female	Schwannoma	Thigh	35
**B. Primary malignant musculoskeletal tumour**			
6/6/59/male	Pleomorphic leiomyosarcoma	Lower back	79
7/6/59/male	Pleomorphic leiomyosarcoma	Lower back	28
8/7/55/male	Myxofibrosarcoma	Thigh	57
9/8/81/male	Pleomorphic leiomyosarcoma	Back	48
10/9/70/male	Myxofibrosarcoma	Shoulder	66
**C. Metastatic musculoskeletal tumour**			
11/10/78/female	AC from gastric cancer	Thigh (muscle)	79
12/11/71/male	SCC from hypopharyngeal cancer	Neck (muscle)	21
13/11/71/male	SCC from hypopharyngeal cancer	Neck (muscle)	28
14/11/71/male	SCC from hypopharyngeal cancer	Neck (muscle)	23
**D. Malignant lymphoma**			
15/12/54/female	Diffuse large B cell lymphoma	Sacrum	87
16/13/85/female	Diffuse large B cell lymphoma	Thigh	57
17/13/85/female	Diffuse large B cell lymphoma	Thigh	20

*AC* adenocarcinoma, *SCC* squamous cell carcinoma

The 17 lesions were assigned to 4 groups: BMSLs (*n* = 5, 29%), PMMSTs (*n* = 5, 29%), MMSTs (*n* = 4, 24%), and MLs (*n* = 3, 18%). The tumour sizes ranged from 20 to 245 mm, with most lesions < 70 mm in long-axis diameter. There were no significant differences in tumour size among groups (*p* = 0.697).

### Standardised uptake values and retention index of maximum SUV

**Fig. 1:**
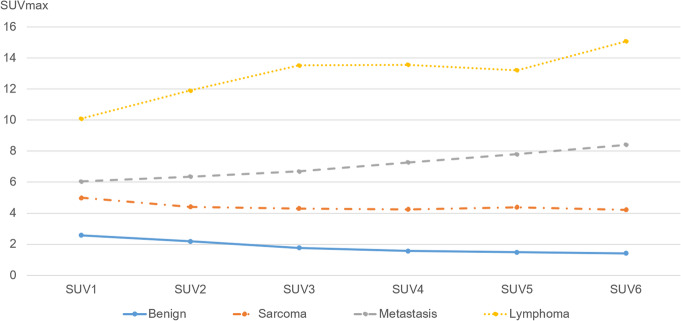
The change in median SUVmax values for each time phase in benign and malignant musculoskeletal lesions

SUVmax, RI-SUV values, and SUVmean are summarised in Tables 2 and 3. Figure 1 shows the change in median SUVmax values for each time phase per study group. SUVmax values decreased gradually or remained relatively stable with minimal fluctuations in all BMSL cases and four of five PMMST cases. In contrast, SUVmax values progressively increased over time in one PMMST case (case no. 9), in which round and polymorphic tumour cells are more densely growing compared with the other two pleomorphic leiomyosarcomas with myxomatous components (case no. 6 and no. 7) and all MMST and ML cases.

**Table 2:** SUVmax and RI-SUV at each time point in each group

Group	BMSL	MMSL	PMMST	MMST	ML	MMST and ML
**SUV1**	2.58 ± 0.50 (1.13–3.73)	6.04 ± 0.66 (4.36–11.42)	5.00 ± 0.75 (4.36–8.51)	6.04 ± 0.49 (4.86–8.51)	10.09 ± 1.12 (7.59–11.42)	7.15 ± 0.90 (4.86–11.42)
**SUV2**	2.18 ± 0.42 (1.07–3.41)	6.35 ± 0.89 (4.19–13.45)	4.41 ± 1.03 (4.19–9.58)	6.35 ± 0.53 (5.63–8.09)	11.91 ± 1.34 (8.90–13.45)	8.09 ± 1.13 (5.63–13.45)
**SUV3**	1.77 ± 0.34 (1.00–2.97)	6.69 ± 1.05 (3.57–14.90)	4.30 ± 1.11 (3.57–9.66)	6.69 ± 0.63 (6.33–9.03)	13.52 ± 1.85 (8.80–14.90)	8.80 ± 1.31 (6.33–14.90)
**SUV4**	1.56 ± 0.32 (1.01–2.86)	7.27 ± 1.08 (3.53–14.80)	4.25 ± 1.22 (3.53–10.13)	7.27 ± 0.89 (6.23–10.28)	13.57 ± 1.59 (9.55–14.80)	9.55 ± 1.25 (6.23–14.80)
**SUV5**	1.49 ± 0.27 (1.11–2.62)	7.81 ± 1.28 (3.38–18.49)	4.39 ± 1.33 (3.38–10.72)	7.81 ± 0.88 (6.97–10.94)	13.21 ± 2.44 (10.14–18.49)	10.14 ± 1.53 (6.97–18.49)
**SUV6**	1.41 ± 0.30 (1.03–2.75)	8.41 ± 1.26 (3.47–17.21)	4.22 ± 1.24 (3.47–10.23)	8.41 ± 0.84 (7.63–11.34)	15.07 ± 2.23 (9.72–17.21)	9.72 ± 1.40 (7.63–17.21)
**RI-SUV**	− 26.27 ± 12.55 (− 50.00 to 23.6)	30.01 ± 9.34 (− 28.89 to 70.57)	− 7.11 ± 8.06 (− 28.89 to 20.21)	48.35 ± 7.29 (34.57–63.79)	31.96 ± 13.57 (28.06–70.57)	38.10 ± 6.53 (28.06–70.57)

The data represent the median ± standard error of the mean; data in parentheses are the ranges

*SUV* standardised uptake value, *RI-SUV* retention index of maximum standardised uptake value, *BMSL* benign musculoskeletal lesion, *MMSL* malignant musculoskeletal lesion, *PMMST* primary malignant musculoskeletal tumour, *MMST* metastatic musculoskeletal tumour, *ML* malignant lymphoma

**Table 3:** SUVmean at each time point in each group

Group	BMSL	MMSL	PMMST	MMST	ML	MMST and ML
**SUV1**	1.13 ± 0.30 (0.75–1.53)	3.69 ± 0.39 (2.55–6.21)	2.75 ± 0.46 (2.55–4.96)	3.69 ± 0.72 (2.92–6.21)	5.21 ± 0.49 (4.32–6.03)	4.32 ± 0.48 (2.92–6.21)
**SUV2**	1.02 ± 0.24 (0.70–1.41)	3.85 ± 0.46 (2.27–6.71)	2.56 ± 0.59 (2.27–5.39)	3.85 ± 0.80 (3.12–6.71)	5.43 ± 0.40 (5.09–6.43)	5.09 ± 0.52 (3.12–6.71)
**SUV3**	1.01 ± 0.21 (0.78–1.34)	3.99 ± 0.51 (2.02–7.01)	2.56 ± 0.64 (2.02–5.53)	3.99 ± 0.74 (3.64–6.83)	6.01 ± 0.56 (5.08–7.01)	5.08 ± 0.53 (3.64–7.01)
**SUV4**	0.94 ± 0.17 (0.77–1.34)	4.23 ± 0.54 (1.95–7.16)	2.47 ± 0.69 (1.95–5.68)	4.23 ± 0.74 (3.60–6.89)	5.98 ± 0.48 (5.56–7.16)	5.56 ± 0.53 (3.60–7.16)
**SUV5**	0.94 ± 0.15 (0.71–1.25)	4.51 ± 0.56 (1.86–7.17)	2.60 ± 0.74 (1.86–5.94)	4.51 ± 0.72 (3.99–7.17)	6.06 ± 0.38 (6.03–7.17)	6.03 ± 0.50 (3.99–7.17)
**SUV6**	0.97 ± 0.18 (0.71–1.28)	4.74 ± 0.60 (1.85–7.72)	2.45 ± 0.73 (1.85–5.87)	4.74 ± 0.76 (4.39–7.65)	6.49 ± 0.58 (5.73–7.72)	5.73 ± 0.53 (4.39–7.72)

The data represent the median ± standard error of the mean; data in parentheses are the ranges

*SUV* standardised uptake value, *BMSL* benign musculoskeletal lesion, *MMSL* malignant musculoskeletal lesion, *PMMST* primary malignant musculoskeletal tumour, *MMST* metastatic musculoskeletal tumour, *ML* malignant lymphoma

All metabolic parameters were significantly higher in MMSLs than in BMSLs (*p* < 0.001 for all SUVmax; *p* = 0.0194 for RI-SUV). The SUVmax values for each time phase were significantly higher for PMMST than for BMSL (*p* = 0.00471 for all SUVmax values). In contrast, no significant differences were detected in RI-SUV between PMMSTs and BMSLs (*p* = 0.78327) or in all parameters between other single groups.

**Fig. 2:**
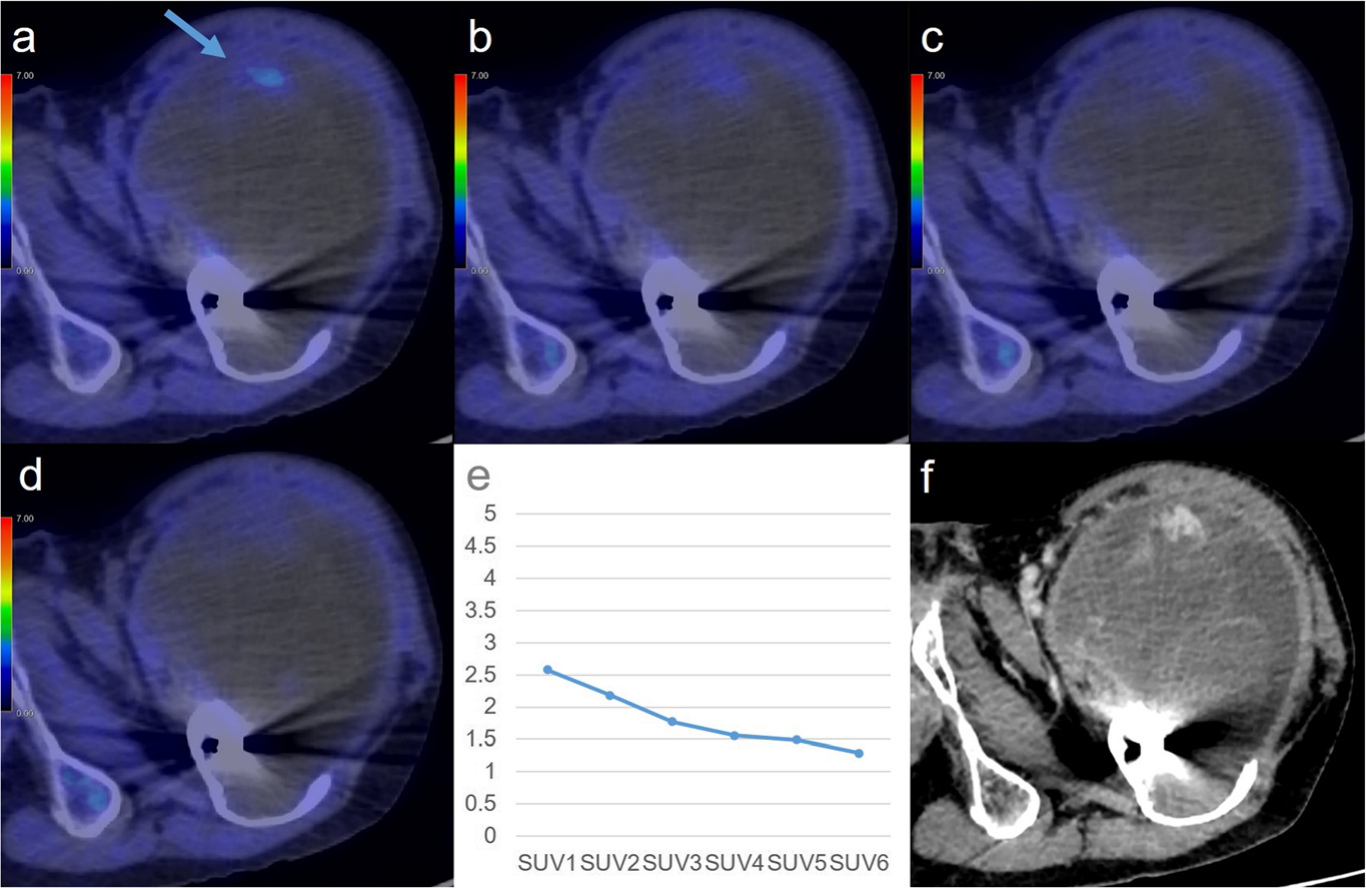
A 67-year-old man with chronic expanding haematoma in the right femur. Dynamic positron-emission tomography/computed tomography (PET/CT) (**a**–**d**) images are shown. In the dynamic first phases (**a**), the focal faint accumulation is identifiable in the anterior part from the dynamic first phase (arrows, SUVmax: dynamic first phase, 2.58). After the dynamic second phases (**b**–**d**), the accumulation gradually becomes subtler in most parts of the tumour (SUVmax: dynamic second phase (**b**), 2.18; dynamic third phase, 1.77; dynamic fourth phase (**c**), 1.56; dynamic fifth phase, 1.49; dynamic sixth phase (**d**), 1.29). The time-SUVmax curve (**e**) shows a low level in SUVmax at the dynamic first phase and a gradual decrease during the dynamic phases. The post-contrast CT with iodine contrast medium (**f**) reveals the enhancing solid component in the anterior part of the haematoma and cortical erosion of thigh bone by the haematoma

**Fig. 3.**
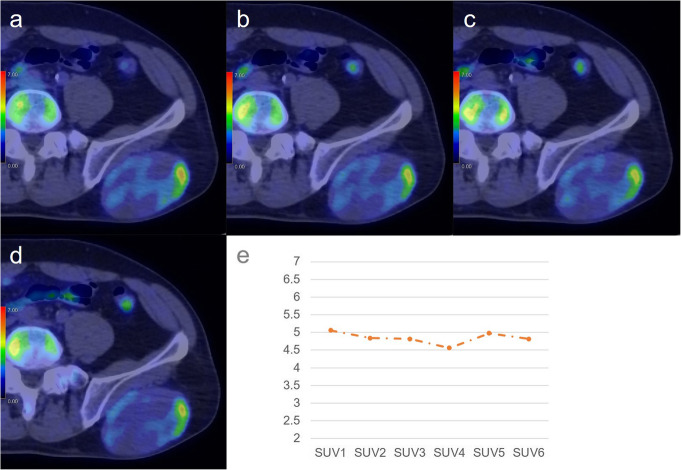
A 59 year-old-man with pleomorphic leiomyosarcoma (grade 2) in the lower back. Dynamic positron-emission tomography/computed tomography (PET/CT) images are shown for the dynamic phases (**a**–**d**). In the dynamic first phases (**a**), the methionine accumulation in soft tissue tumours is inhomogeneous, and the focal intense accumulation is identifiable in the left portion of the tumour (SUVmax, 5.06). After the dynamic second phase (**b**–**d**), the focal intense accumulation gradually decreased (SUVmax: dynamic second phase (**b**), 4.84; dynamic third phase, 4.82; dynamic fourth phase (**c**), 4.56; dynamic fifth phase, 4.98; dynamic sixth phase (**d**), 4.82). The SUVmax remained relatively stable with minimal fluctuation during the dynamic phases (**e**)

**Fig. 4:**
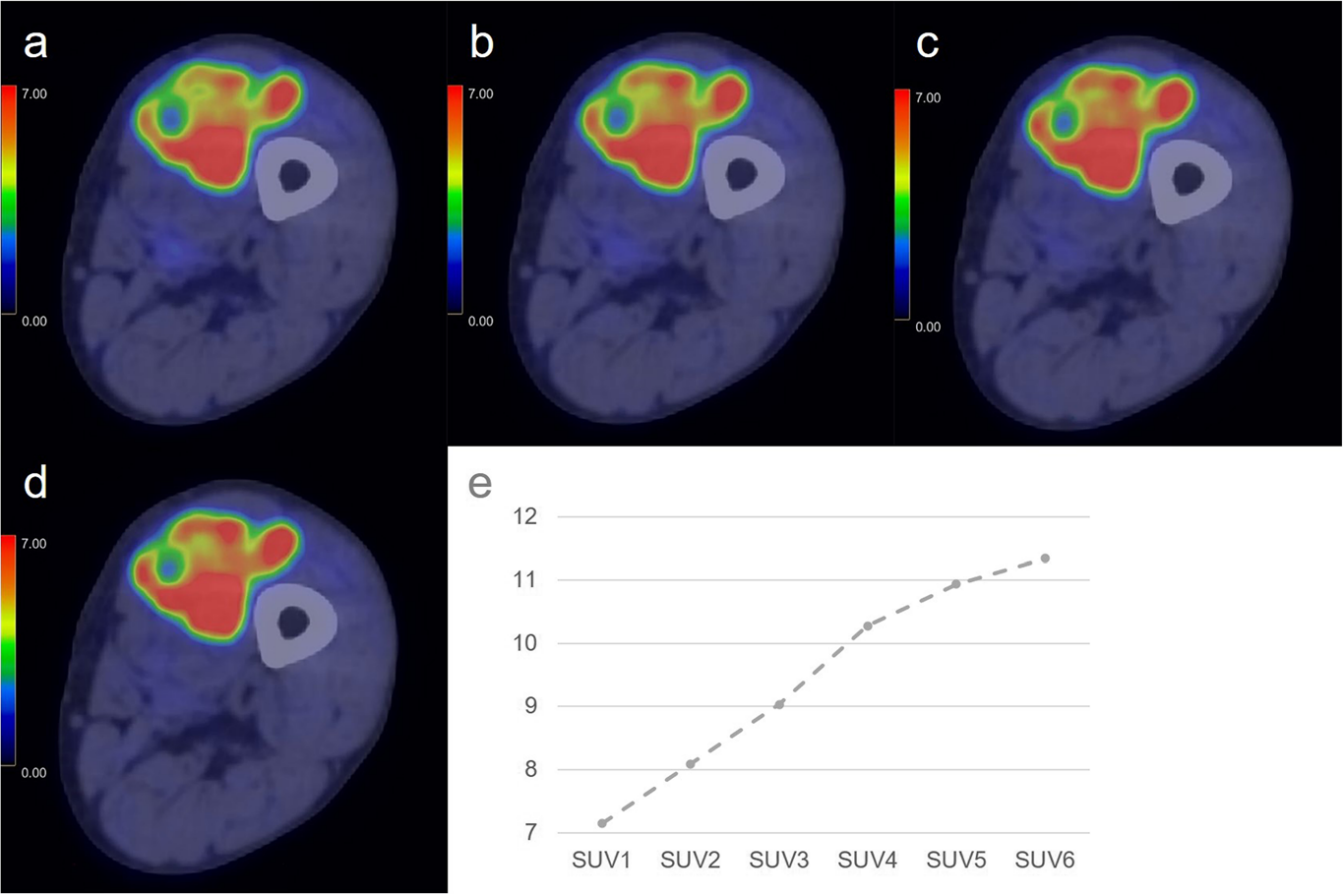
A 78-year-old woman with metastatic soft tissue tumour from gastric cancer in the left thigh. Dynamic positron-emission tomography/computed tomography (PET/CT) (**a**–**d**) images are shown. In all dynamic phases (**a**–**d**), the soft tissue tumour is avid for methionine (MET). The inhomogeneous and intense MET accumulation in soft tissue tumours is identifiable from the dynamic first phase (SUVmax: dynamic first phase (**a**), 7.15; dynamic second phase (**b**), 8.09; dynamic third phase, 9.03; dynamic fourth phase (**c**), 10.28; dynamic fifth phase, 10.94; dynamic sixth phase (**d**), 11.34). The accumulation gradually becomes more intense in most parts of the tumour during the dynamic phases (**a**–**d**). The time-SUVmax curve (**e**) shows a high level in SUVmax at the dynamic first phase and a continuous increase during the dynamic phases

**Fig. 5:**
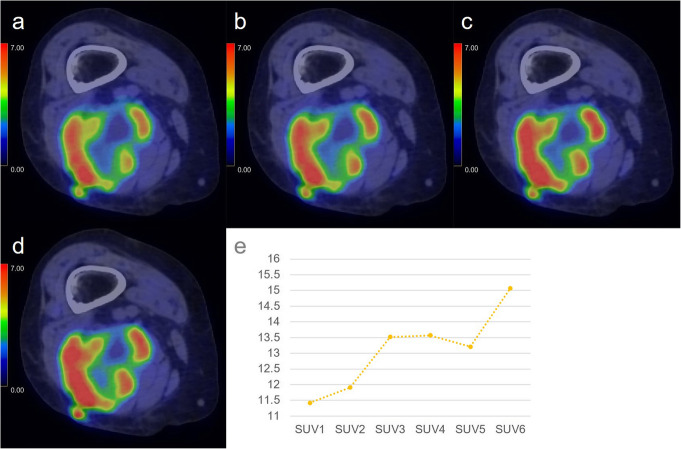
An 85-year-old woman with diffuse large B cell lymphoma in the right thigh. Dynamic positron-emission tomography/computed tomography (PET/CT) (**a**–**d**) images are shown. In all dynamic phases (**a**–**d**), methionine accumulation in the soft tissue tumour is inhomogeneous, and intense accumulation is identifiable in the peripheral regions from the dynamic first phase (SUVmax: dynamic first phase (**a**), 11.42; dynamic second phase (**b**), 11.91; dynamic third phase, 13.52; dynamic fourth phase (**c**), 13.57; dynamic fifth phase, 13.21; dynamic sixth phase (**d**), 15.07). The accumulation gradually becomes more intense in most parts of the tumour during the dynamic phases (**a**–**d**). The time-SUVmax curve (**e**) shows a high level in SUVmax at the dynamic first phase (5–15 min after injection) and a gradual increase during the dynamic phases

Values of all metabolic parameters were significantly higher for non-PMMST-malignant tumours (i.e. MMSTs and MLs) than for BMSL (each *p* value = 0.01246). RI-SUV values were significantly higher for non-PMMST-malignant tumours than for PMMSTs (*p* = 0.01246). Figures 2, 3, 4, and 5 show representative dynamic ^11^C-MET PET/CT images of each pathology.

### ROC curves and cut-off values for SUVmax and RI-SUV

Table 3 shows the threshold (i.e. cut-off) values for each parameter and AUC for discriminating the groups. For most comparisons between groups, cut-off SUVmax values for each dynamic phase and cut-off RI-SUV yielded a sensitivity of 100.0%, specificity of 100.0%, PPV of 100.0%, NPV of 100.0%, and accuracy of 100.0% with AUCs of 1.00 (cut-off SUV value: MMSL from BMSL: SUV1 4.36, SUV2 4.19, SUV3 3.57, SUV4 3.53, SUV5 3.38, SUV6 3.47, RI-SUV − 7.11; PMST from BMSL: SUV1 4.36, SUV2 4.19, SUV3 3.57, SUV4 3.53, SUV5 3.38, SUV6 3.47; MMST and ML from BMSL: SUV1 4.86, SUV2 5.63, SUV3 6.33, SUV4 6.23, SUV5 6.97, SUV6 7.63, RI-SUV 28.06; MMST and ML from PMMST: RI-SUV 28.06). For differentiating MMSLs and BMSLs, the cut-off RI-SUV value yielded a sensitivity of 83.3%, specificity of 80.0%, PPV of 90.9%, NPV of 66.7%, and accuracy of 82.4% with an AUC of 0.8667. Delong’s test did not reveal any significant differences between the AUCs for all parameters (all *p* > 0.05).

## Discussion

The present study identified different dynamic changes in maximum ^11^C-MET uptake depending on MSL histology. In all BMSL and most PMMST cases, SUVmax decreased gradually with time or remained relatively stable with minimal fluctuations. In contrast, SUVmax progressively increased over time in all MMST and ML cases and one PMMST case. In a previous study using ^11^C-MET dynamic PET in small animals (Zhao et al., 2011), ^11^C-MET uptake occurred faster and at a higher level in granulomas than in tumours. Further, the washout of ^11^C-MET from the granuloma and continuous accumulation of ^11^C-MET in the tumour were observed at later time points. In the report on a brain tumour, dynamic changes in ^11^C-MET uptake varied between different pathologies and a significant dynamic decrease was observed in the max MET tumour-to-normal ratio in meningiomas and oligodendrocytic tumours, whereas a significant dynamic increase was observed in glioblastomas and MLs (Aki et al., 2012)*.* In another report, Nomura et al. demonstrated MET-SUVmax increased over time during the late phase on 35-min dynamic scans in primary central nervous system lymphomas (Nomura et al., 2018). These findings could be attributed to the differences in local blood flow, stagnation of MET in the vascular beds of lesions, biological activity, cellular proliferation activity, transporters of amino acids, and enzyme activity within each lesion, degree of neovascularisation (Zhao et al., 2011; Aki et al., 2012; Okada et al., 2012). Taken together, we speculated that the strong angiogenesis and proliferation activity and the active amino acid transport mechanism or the transmethylation pathway might affect the high uptake of MET to MMST and ML in the early dynamic phase and might lead to the difference in the MET uptake to BMSL. PMMST sarcomas are composed of mesenchymal elements that contain various amounts of relatively metabolically inactive tissue such as myxoid ground substance, osteoid tissue, fluid, and necrotic tissue. They also contain a population showing varying cellularity, tissue types, and levels of dedifferentiation (Zhang et al., 2004). These might have led to the various MET uptake patterns in the present study. Therefore, the dynamic uptake patterns of ^11^C-MET in MSLs may reveal additional information about histological and biological characteristics of different MSLs, which cannot be obtained from morphologic and static PET imaging and may be helpful for the differential diagnosis of MSLs. However, further investigation is required to reveal the mechanism and the efficiency of ^11^C-MET PET/CT in patients with MSL lesions.

In this study, we observed that SUVmax values for each time phase were significantly higher for MMSL and PMMST than for BMSL. RI-SUV also differed significantly between BMSL and MMSL. Cut-off SUVmax values for each dynamic phase could have high diagnostic capacity in this study population, although the cut-off values in the present study are not generalisable to other institutions in clinical practice. In contrast, no significant differences were detected in other parameters between the other single groups, probably due to the small study population (four MMSL and three ML cases). However, values of all metabolic parameters were significantly higher in non-PMMST-malignant tumours (four MMST and three ML cases) than in BMSLs. RI-SUV was also significantly higher in non-PMMST-malignant tumours than in PMMSTs. A correlation between histological grade and increased MET uptake has been reported for brain, lung, and uterine tumours, and lymphomas (Derlon et al., 1989; Fujiwara et al., 1989; Lapela et al., 1994; Nuutinen et al., 1998). The MET uptake reflects the high need for amino acids for protein synthesis in cancer cells as well as enhanced transmethylation reaction in neoplastic tissue (Nuutinen et al., 1998). These results suggested that ^11^C-MET uptake in MSLs can differ depending on MSL histology, and ^11^C-MET uptake in MMSTs from adenocarcinoma and squamous cell carcinoma as well as ML (diffuse large B-cell lymphoma) can be more intense compared to that in BMSLs and PMMSTs in this study population. In addition, dynamic ^11^C-MET PET/CT may have the diagnostic power to discriminate the histology of primary unknown MSLs with semiquantitative analyses. Dynamic ^11^C-MET PET/CT may have the potential to aid prediction of histopathology in cases where percutaneous biopsy cannot be performed or where there are multiple sites with various suspected morphological subtypes. Moreover, recent studies have reported the efficacy of recombinant methioninase combined with palbociclib or caffeine and doxorubicin for sarcomas (Igarashi et al., 2018; Higuchi et al., 2018). Dynamic ^11^C-MET PET/CT could be a possible biomarker for metabolic therapeutic targets in PMMSTs.

Our prospective study was limited by the small number of patients included and did not include granulomas, which show MET-avidity (false-positive) on early dynamic phases. Further, we did not assess the pathological background responsible for the difference in MET uptake into MSLs or involvement of L-type amino acid transporter 1 in the tumours, nor did we perform kinetic modelling in MET-PET. A multi-institutional trial using a larger patient population that includes each histological subtype combined with immunohistochemical analysis may provide a clearer picture and more comprehensively reveal the diagnostic capacity of dynamic ^11^C-MET PET/CT scans for MSL. Despite its limitations, the present study identified various dynamic changes in maximum ^11^C-MET uptake depending on the histology of the MSL and demonstrated the high diagnostic ability of this approach for the differential diagnosis of primary unknown MSLs in clinical practice with optimal cut-off levels of SUVmax and RI-SUV. Moreover, our dynamic ^11^C-MET PET/CT analysis can be easily obtained from dynamic PET studies with list-mode in recent clinical settings. It can be routinely performed clinically without the need for additional invasive methods, such as continuous phlebotomy for arterial input function.

## Conclusion

The present study demonstrated a gradual decrease or stability with time in ^11^C-MET uptake in BMSLs and most PMMSTs, and a transitional increase in ^11^C-MET uptake in MMSTs and MLs. In addition, MET-avidity in MMSL was stronger than that in BMSL during all dynamic phases. The dynamic uptake patterns of ^11^C-MET in MSLs may provide additional information about the histological and biological characteristics of different MSLs. Moreover, dynamic ^11^C-MET PET/CT had extremely high diagnostic accuracy for differentiation of MSL using semiquantitative analyses of SUVmax and RI-SUV in the current patient population. Additional studies with more patients are needed to determine which dynamic phase is superior for differentiation in MSL.

## Data Availability

The image datasets generated and/or analysed during the current study are not publicly available due to data protection guidelines but are available from the corresponding author on reasonable request.

## Abbreviations

^11^C-MET: Carbon-11 methionine
AUC: Area under the curve
BMSL: Benign musculoskeletal lesion
CT: Computed tomography
ML: Malignant lymphoma
MMSL: Malignant musculoskeletal lesions
MMST: Metastatic musculoskeletal tumour
MSL: Musculoskeletal lesion
NPV: Negative predictive value
PET: Positron-emission tomography
PMMST: Primary malignant musculoskeletal tumour
PPV: Positive predictive value
RI-SUV: Retention index for dynamic scans
ROC: Receiving operating characteristic
SUVmax: Maximum standardised uptake values
SUVmean: Mean standardised uptake values
VOI: Volume of interest
